# Dose-Dependent Osteoinduction by rhBMP-2-Loaded β-Tricalcium Phosphate Scaffolds in Rabbit Critical-Sized Calvarial Defects: Histological, Histomorphometric, CD31 Immunohistochemical Evaluation

**DOI:** 10.3390/ijms27083609

**Published:** 2026-04-18

**Authors:** Solaf Abdulqadir Mustafa, Chenar Anwar Mohammad, Rafal Abdulrazaq Alrawi

**Affiliations:** 1 Department of Periodontics, College of Dentistry, Hawler Medical University, Erbil 44001, Iraq; chenar.anwar@hmu.edu.krd; 2 Department of Clinical Analysis, College of Pharmacy, Hawler Medical University, Erbil 44001, Iraq; rafal.alrawi@hmu.edu.krd

**Keywords:** β-tricalcium phosphate, bone morphogenetic protein-2, critical size defect, immunohistochemistry, rabbit calvaria, osteogenesis

## Abstract

Critical-sized bone defects represent a major clinical challenge, as defects of this magnitude do not heal spontaneously without regenerative intervention. This study aimed to evaluate the osteoinductive effects of recombinant human bone morphogenetic protein-2 (rhBMP-2) loaded β-tricalcium phosphate (β-TCP) scaffolds on bone regeneration and vascularization in a rabbit calvarial critical-sized defect model. Eighteen male New Zealand White rabbits were used, and four standardized circular defects (5 mm in diameter) were created in the calvaria of each animal. The defects were assigned to four groups: control (unfilled), β-TCP + 5 µg rhBMP-2, β-TCP + 10 µg rhBMP-2, and β-TCP + 20 µg rhBMP-2. Bone healing was evaluated at 2, 4, and 8 weeks using histological, histomorphometric, and cluster of differentiation 31 (CD31) immunohistochemical analyses. The results demonstrated that rhBMP-2–loaded β-TCP scaffolds significantly enhanced bone regeneration compared with the control group, with a progressive increase in bone formation observed with increasing rhBMP-2 doses. The β-TCP + 20 µg rhBMP-2 group exhibited the highest levels of new bone formation, more advanced bone maturation, improved collagen organization, and increased vascularization. However, no statistically significant differences were observed between the 10 µg and 20 µg groups at later time points (*p* > 0.05), suggesting a dose-dependent saturation (plateau) effect. In conclusion, rhBMP-2–loaded β-TCP scaffolds promote bone regeneration and angiogenesis in a dose-related manner up to a threshold, beyond which additional increases in dose do not result in proportional improvements. These findings emphasize that optimal rhBMP-2 dosing is critical to maximize regenerative outcomes while avoiding unnecessary dose escalation.

## 1. Introduction

The dimension of a bone defect is a key determinant influencing its intrinsic regenerative capacity. When a defect surpasses a certain threshold—defined as a critical-sized defect—spontaneous bone healing fails to occur without the intervention of regenerative materials. In such circumstances, where osteoconductive matrices and osteogenic signals are insufficient, bone repair requires the incorporation of bioactive and osteoinductive agents to restore both structure and function [1]. In addition, bone regeneration may vary depending on anatomical site, bone structure, and age, as demonstrated in rabbit models [2].

Autografts continue to represent the standard grafting option, offering both structural support and biological stimulation for bone regeneration [3]. Donor-site morbidity, limited supply, and the need for an additional surgery motivate the search for synthetic, bioactive alternatives [4]. Consequently, calcium-phosphate bioceramics have attracted extensive interest because of their biocompatibility, predictable resorption, and ability to interact with cells and growth factors [5]. Among the various synthetic bioceramics, β-tricalcium phosphate (β-TCP) has attracted particular attention for its proven safety and bone affinity. Its chemical composition and microstructure closely resemble those of natural bone, providing excellent osteoconductivity and biocompatibility, while its controlled biodegradation allows progressive substitution by newly formed bone [6]. Nevertheless, β-TCP sometimes resorbs more rapidly than bone is deposited, producing an imbalance between scaffold loss and tissue regeneration [6].

Although some studies described an inherent osteoinductive capacity of β-TCP, this characteristic remains controversial and appears to vary across animal models, largely depending on the host’s biological environment and circulating growth factors. These limitations have restricted the overall regenerative potential of β-TCP, prompting researchers to enhance its biological functionality by combining it with proteins, drugs, or growth factors [7].

Recombinant human bone morphogenetic protein-2 (rhBMP-2) is widely recognized as a key osteoinductive factor that has been extensively investigated for clinical use. BMPs belong to the transforming growth factor-β (TGF-β) superfamily and were first identified by Urist [8], who observed ectopic bone formation after implantation of demineralized bone matrix [9]. Structurally, BMPs are dimeric proteins of approximately 120 amino acids stabilized by disulfide linkages and containing a heparin-binding region [10]. Functionally, they govern cell proliferation, differentiation, matrix synthesis, and apoptosis. BMP-2 specifically promotes mesenchymal-stem-cell commitment to the osteoblastic lineage, stimulates osteoid deposition, and is clinically approved for use with collagen carriers in orthopedic and dental procedures [9].

Within critical-sized bone defects, BMP-2 serves a central regulatory role in coordinating osteogenesis and angiogenesis while modulating osteoclast activity through the Receptor Activator of Nuclear Factor Kappa-B Ligand (RANKL) pathway [11]. Experimental studies have shown that bone formation rises proportionally with BMP-2 dose until an optimal level is reached, beyond which the response plateaus or declines [11].

Despite the widespread use of rhBMP-2 in bone tissue engineering, the optimal dose required to maximize osteoinductive efficiency while minimizing potential adverse effects remains unclear. Although previous studies have investigated the osteogenic potential of rhBMP-2, few have provided a comprehensive and quantitative evaluation of both osteogenesis and angiogenesis within a standardized critical-sized defect model. This study provides an integrated assessment of osteogenesis, collagen maturation, and angiogenesis using combined histological, histomorphometric, and immunohistochemical analyses. Furthermore, it examines the dose-dependent effects of rhBMP-2 across multiple healing stages and demonstrates that increases in dose beyond a certain threshold do not result in statistically significant additional bone formation, suggesting a plateau-like response. These findings contribute to the optimization of therapeutic dosing strategies in bone regeneration. The aim of this study was to evaluate bone regeneration and vascularization in a rabbit calvarial critical-sized defect model.

## 2. Results

### 2.1. Histological Assessment of Calvarial Bone Defects (H&E, ×400)

**Figure 1:**
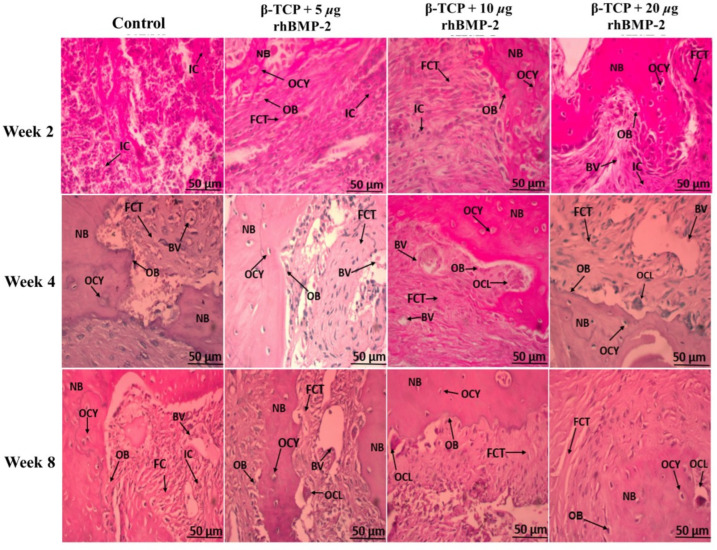
Histological evaluation of rabbit calvarial defects treated with β-TCP loaded with different doses of rhBMP-2 at 2, 4, and 8 weeks. IC, inflammatory cells; FCT, fibrous connective tissue; NB, New bone; BV, blood vessel; OB, Osteoblast; OCY, Osteocyte; OCL, Osteoclast. Scale bar = 50 µm.

Figure 1 shows the histological features of bone regeneration in the experimental groups at different healing intervals. In week two, the control group demonstrated predominantly immature tissue with inflammatory cell infiltration within the defect area. The β-TCP + 5 µg rhBMP-2 group showed fibrous connective tissue occupying the defect region with reduced inflammatory infiltration. In the β-TCP + 10 µg rhBMP-2 group, early bone matrix containing osteocyte lacunae was detected. The β-TCP + 20 µg rhBMP-2 group exhibited early new bone formation surrounded by bone matrix.

In week four, the control group showed fibrous connective tissue with limited bone matrix formation. The β-TCP + 5 µg rhBMP-2 group demonstrated bone matrix containing osteocytes within the defect area. The β-TCP + 10 µg rhBMP-2 group showed a thicker and more continuous bone matrix with visible vascular structures. In the β-TCP + 20 µg rhBMP-2 group, new bone formation with improved tissue organization was observed.

In week eight, the control group showed incomplete healing with persistent fibrous tissue and limited bone matrix formation. The β-TCP + 5 µg rhBMP-2 group demonstrated new bone formation within the defect area. Both the β-TCP + 10 µg rhBMP-2 and β-TCP + 20 µg rhBMP-2 groups exhibited mature bone formation characterized by organized bone matrix and numerous osteocyte lacunae, with the 20 µg rhBMP-2 group showing the most developed and well-organized bone architecture.

### 2.2. Histomorphometric Analysis of Bone Regeneration

#### 2.2.1. New Bone Formation (%)

**Figure 2:**
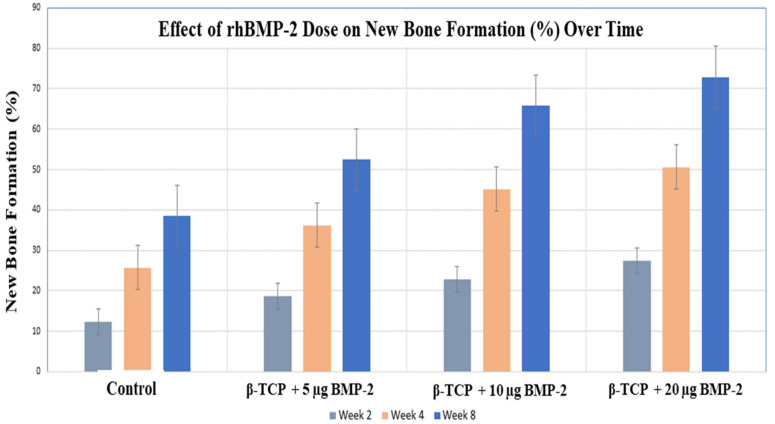
New bone formation (%) in rabbit calvarial defects treated with β-TCP loaded with different doses of BMP-2 at 2, 4, and 8 weeks. Data are presented as mean ± SD.

Quantitative histomorphometric analysis demonstrated a progressive increase in new bone formation across all groups during the healing period (Table 1; Figure 2). The amount of newly formed bone was greater in the rhBMP-2–treated groups compared with the control group and increased with higher rhBMP-2 doses.

In week two, the control group manifested the lowest level of bone formation (12.4 ± 3.1%). In contrast, the experimental groups treated with β-TCP loaded with rhBMP-2 showed higher values. The β-TCP + 5 µg rhBMP-2 group demonstrated 18.6 ± 3.8%, while the β-TCP + 10 µg rhBMP-2 group reached 22.9 ± 4.0%, and the β-TCP + 20 µg rhBMP-2 group showed the highest bone formation at this time point (27.3 ± 4.4%).

In week four, a marked increase in bone regeneration was observed in all groups. The control group showed 25.7 ± 4.2% new bone formation. Higher values were observed in the β-TCP + 5 µg rhBMP-2 group (36.2 ± 4.5%), followed by the β-TCP + 10 µg rhBMP-2 group (45.1 ± 5.2%), while the β-TCP + 20 µg rhBMP-2 group exhibited the greatest bone formation (50.6 ± 5.9%).

In week eight, bone regeneration further increased across all groups. The control group reached 38.5 ± 5.0%, whereas the β-TCP + 5 µg rhBMP-2 group showed 52.4 ± 6.1%. The β-TCP + 10 µg rhBMP-2 group demonstrated 65.8 ± 6.7%, while the β-TCP + 20 µg rhBMP-2 group displayed the highest value of new bone formation (72.9 ± 7.2%).

#### 2.2.2. Inflammatory Response and Granulation Tissue

**Figure 3:**
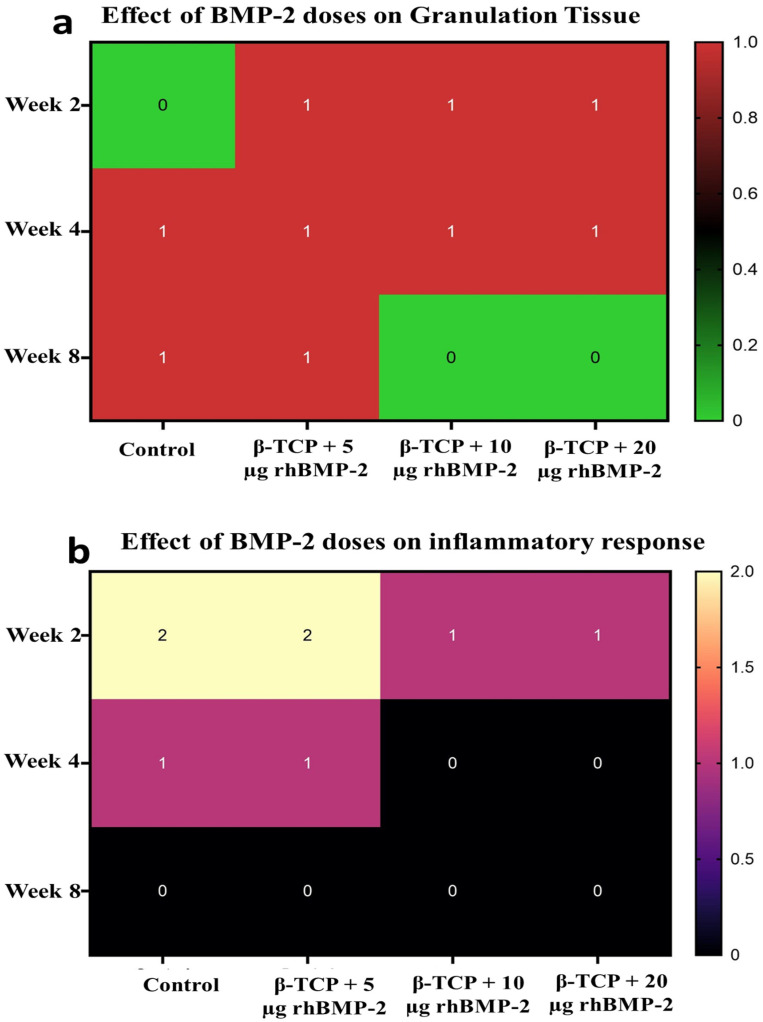
Heatmap showing semi-quantitative analysis of tissue response across groups and time points. (**a**) Granulation tissue formation across different experimental groups at weeks 2, 4, and 8. (**b**) Inflammatory response across different experimental groups at weeks 2, 4, and 8. Heatmap cells represent the dominant semi-quantitative score category for each group and time point, whereas the corresponding mean ± standard deviation (SD) values are provided in the text.

In week two, the control group indicated the highest inflammatory reaction (2.33 ± 0.52), whereas rhBMP-2-treated groups presented lower mean scores that decreased progressively with increasing rhBMP-2 dosage, 1.67 ± 0.52 for (β-TCP + 5 µg rhBMP-2) 1.33 ± 0.52 for (β-TCP + 10 µg rhBMP-2), and 1.00 ± 0.00 for (β-TCP + 20 µg rhBMP-2). Granulation tissue formation was absent in the control group (0.00 ± 0.00) but evident in the BMP-2-treated groups, with mild to moderate fibro-angiogenic tissue particularly in the (β-TCP + 5 µg rhBMP-2) group (1.33 ± 0.52), as illustrated in Figure 3a,b.

In week four, inflammatory scores declined across all groups, reaching 1.25 ± 0.50 in the control group and stabilizing at 1.00 ± 0.00 in the rhBMP-2-treated groups. Granulation tissue scores remained mild and consistent among rhBMP-2 groups (1.00 ± 0.00), while the control group exhibited slightly higher values (1.33 ± 0.52) as illustrated in Figure 3a,b.

In week eight, inflammatory activity was minimal in all groups, with complete absence in the (β-TCP + 10 µg rhBMP-2 and β-TCP + 20 µg rhBMP-2) groups (0.00 ± 0.00). Granulation tissue formation showed a further reduction, with mean scores of 1.33 ± 0.52 in the control group, 1.00 ± 0.00 in the (β-TCP + 5 µg rhBMP-2) group, 0.33 ± 0.52 in the (β-TCP + 10 µg rhBMP-2) group, and 0.00 ± 0.00 in the (β-TCP + 20 µg rhBMP-2) group, as illustrated in Figure 3a,b.

### 2.3. Quantitative Analysis of Bone Cell Counts

The mean (±SD) numbers of osteocytes, osteoblasts, and osteoclasts varied among groups across the healing intervals, with similar values observed between 4 and 8 weeks in several groups.

**Figure 4:**
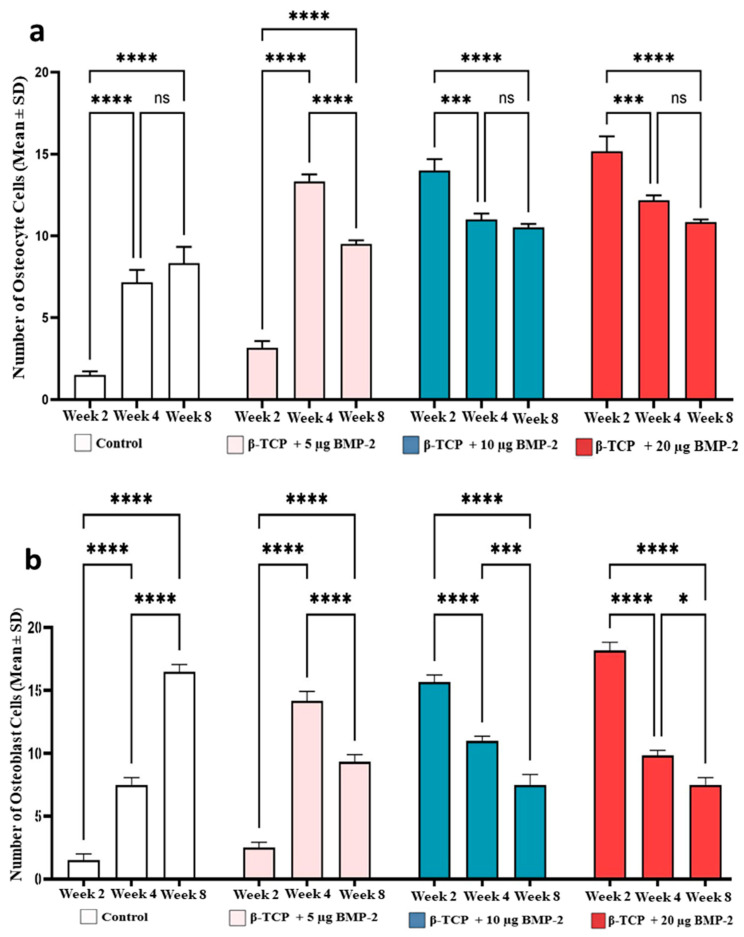

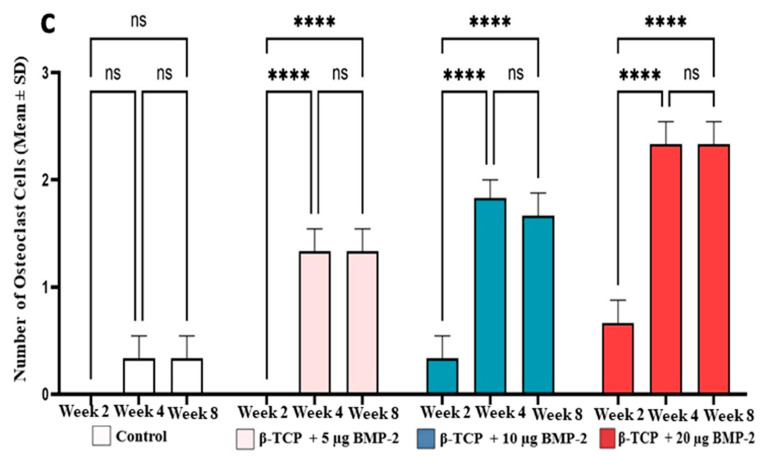
Quantitative analysis of bone cell populations during healing of rabbit calvarial defects treated with different doses of rhBMP-2 loaded β-TCP scaffolds. (**a**) Number of osteocyte cells (mean ± SD), (**b**) number of osteoblast cells (mean ± SD), and (**c**) number of osteoclast cells (mean ± SD) in the defect sites in week two, four and eight postoperatively. Four experimental groups were evaluated: control, β-TCP + 5 µg rhBMP-2, β-TCP + 10 µg rhBMP-2, and, β-TCP + 20 µg rhBMP-2. Bars represent mean values with standard deviation. Statistical significance between groups and time points is indicated as follows: * *p* < 0.05, *** *p* < 0.001, **** *p* < 0.0001; ns indicates non-significant differences.

Osteocytes: In the control group, osteocyte counts increased significantly from week two to week four and eight (*p* < 0.0001), while the difference between week four and eight was not statistically significant. In the β-TCP + 5 µg rhBMP-2 group, osteocyte numbers increased significantly in week four compared with week two (*p* < 0.0001) and decreased in week eight, although the values remained significantly higher than those in week two (*p* < 0.0001). In the β-TCP + 10 µg rhBMP-2 and β-TCP + 20 µg rhBMP-2 groups, osteocyte counts were highest in week two and decreased significantly at 4 and 8 weeks (*p* < 0.001), with no significant difference between 4 and 8 weeks (Figure 4a).

Osteoblasts: In the control group, osteoblast counts increased significantly from week two to week four (*p* < 0.0001) and further increased in week eight (*p* < 0.0001). In the β-TCP + 5 µg rhBMP-2 group, osteoblast numbers increased significantly in week four compared with week two (*p* < 0.0001) and decreased in week eight, although remaining significantly higher than those in week two (*p* < 0.0001). In the β-TCP + 10 µg rhBMP-2 and β-TCP + 20 µg rhBMP-2 groups, osteoblast counts were highest in week two and decreased significantly at 4 and 8 weeks (*p* < 0.0001) (Figure 4b).

Osteoclasts: In the control group, osteoclast counts remained low in week two, four and eight with no statistically significant differences. In the β-TCP + 5 µg rhBMP-2 group, osteoclast numbers increased significantly in week four compared with week two (*p* < 0.0001) and remained similar in week eight. In the β-TCP + 10 µg rhBMP-2 and β-TCP + 20 µg rhBMP-2 groups, osteoclast counts increased significantly in week four and eight compared with week two (*p* < 0.0001), with no significant difference between 4 and 8 weeks (Figure 4c).

### 2.4. Special Stains—Masson’s Trichrome

**Figure 5:**
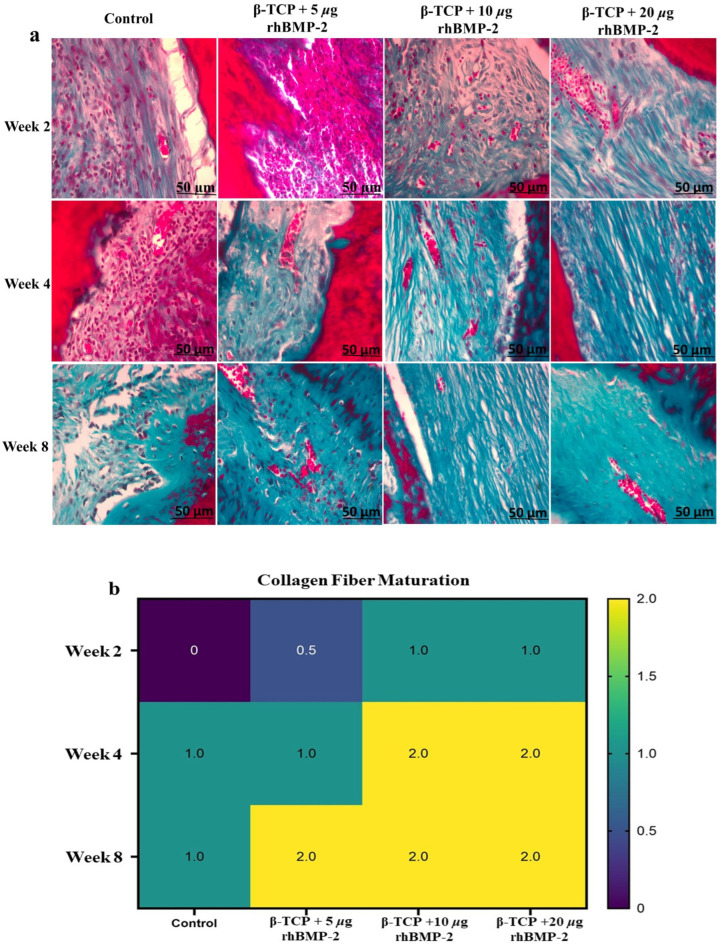
Masson’s Trichrome Analysis of Collagen Deposition During Bone Healing. (**a**) Representative sections showing collagen deposition and maturation in control and β-TCP + rhBMP-2 groups (5, 10, and 20 µg) at 2, 4, and 8 weeks. Collagen fibers appear blue/green, indicating progressive tissue organization. (**b**) Heatmap illustrating semi-quantitative collagen maturation across groups. Scale bar = 50 µm.

Masson’s trichrome staining demonstrated progressive collagen deposition and matrix maturation in all experimental groups during the healing period, as illustrated in Figure 5a,b.

In week two, the control group predominantly showed immature collagen fibers arranged in a loose reticular pattern (Score 0). In contrast, the β-TCP + 5 µg rhBMP-2 group exhibited moderately organized collagen fibers (Score 1). The β-TCP + 10 µg rhBMP-2 and β-TCP + 20 µg rhBMP-2 groups also demonstrated moderately organized collagen fibers (Score 1).

In week four, the control group presented moderately organized collagen bundles (Score 1) with limited new bone formation. The β-TCP + 5 µg rhBMP-2 group showed similar collagen organization. In contrast, the β-TCP + 10 µg rhBMP-2 and β-TCP + 20 µg rhBMP-2 groups displayed dense and more organized collagen bundles (Score 2).

In week eight, the control group continued to show moderately organized collagen fibers (Score 1). In comparison, all rhBMP-2–treated groups demonstrated dense, mature collagen bundles (Score 2). The β-TCP + 20 µg rhBMP-2 group exhibited the most advanced collagen organization.

### 2.5. CD31 Immunohistochemical Analysis

**Figure 6:**
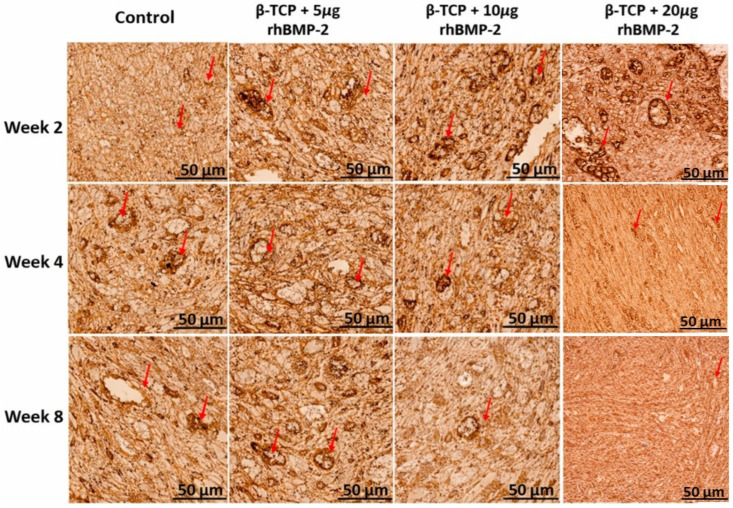
Immunohistochemical staining of CD31 demonstrating vascularization within bone defects across different experimental groups at 2, 4, and 8 weeks post-surgery. CD31-positive endothelial cells, indicative of newly formed blood vessels, are highlighted by brown staining. Red arrows indicate areas of positive CD31 expression, representing vascular structures within the regenerating tissue. Scale bar = 50 µm.

Representative CD31 immunohistochemical staining demonstrated differences in vascular expression among the experimental groups during the healing period (Figure 6), and these findings were supported by quantitative analysis of the CD31-positive vascular area (%) (Table 2).

In week two, the control defects exhibited relatively low CD31-positive staining, with a mean vascular area of 18.0 ± 3.0%**.** In contrast, all β-TCP + rhBMP-2 groups showed markedly increased vascular expression in a dose-dependent manner, with mean values of 35.0 ± 4.2% in the β-TCP + 5 µg rhBMP-2 group, 49.0 ± 5.0% in the β-TCP + 10 µg rhBMP-2 group, and 57.0 ± 5.5% in the β-TCP + 20 µg rhBMP-2 group.

In week four, CD31 expression increased in the control group to 35.0 ± 4.5%**,** representing the highest vascular area among all groups. In contrast, the β-TCP + rhBMP-2 groups demonstrated lower values compared with control, with mean vascular areas of 32.0 ± 4.0% in the 5 µg rhBMP-2 group, 28.0 ± 3.8% in the 10 µg rhBMP-2 group, and 25.0 ± 3.5% in the 20 µg rhBMP-2 group.

In week eight, CD31-positive staining decreased in all groups compared with week 4. The control group showed a mean vascular area of 25.0 ± 3.8%**,** followed by 22.0 ± 3.5% in the β-TCP + 5 µg rhBMP-2 group, 18.0 ± 3.0% in the β-TCP + 10 µg rhBMP-2 group, and 15.0 ± 2.8% in the β-TCP + 20 µg rhBMP-2 group.

## 3. Discussion

Bone morphogenetic protein-2 (BMP-2) is widely recognized as one of the most potent osteoinductive growth factors involved in bone regeneration. It has been extensively investigated in bone tissue engineering because of its ability to stimulate osteoblast differentiation, extracellular matrix production, and new bone formation [12]. However, previous experimental and clinical studies have reported that excessively high rhBMP-2 doses may lead to adverse effects such as soft-tissue swelling, inflammatory reactions, and ectopic bone formation. These findings highlight the importance of determining an optimal and safe dosage for clinical applications [13]. Although several studies have reported potential adverse effects associated with high doses of rhBMP-2, such as ectopic bone formation and increased inflammatory reactions, no evidence of abnormal bone formation or severe inflammatory response was observed in the present study. However, the evaluation of such adverse effects was limited to histological assessment and should be further investigated in future studies.

The rabbit calvarial model is commonly used in experimental bone regeneration studies because it is surgically accessible, provides a stable cortical surface, and allows reliable evaluation of biomaterials and osteogenic growth factors [12]. Several studies have demonstrated that bone regeneration in rabbits is influenced by anatomical location and bone microstructure [2], while standardized surgical models further support the reliability of this animal model in bone research [14]. In such models, critical-size defects (CSDs) are created to properly evaluate regenerative materials. These defects are defined as bone defects that do not heal spontaneously during the lifetime of the animal without intervention [15,16].

In rabbit calvarial models, circular defects ranging from 5–8 mm are commonly used to evaluate bone healing [17]. In the present study, 5 mm calvarial defects were selected because they allow reliable assessment of bone regeneration while maintaining structural stability of the calvarial bone. Therefore, this study investigated the regenerative potential of three rhBMP-2 doses (5 µg, 10 µg, and 20 µg) delivered through β-TCP scaffolds to determine the most effective dose for enhancing bone regeneration in calvarial defects. However, it should be noted that the use of relatively small defects may create a more favorable biological environment for bone healing compared to large clinical defects. Moreover, although the animals were classified as adults based on age and weight, they may not have reached full skeletal maturity, which is associated with enhanced regenerative capacity. Therefore, the outcomes observed in this model may be overestimated relative to clinical conditions, and caution should be exercised when extrapolating these findings to larger defects in fully mature human patients.

Histopathological evaluation demonstrated clear differences in bone regeneration between the control and rhBMP-2–treated groups. The control defects showed limited osteoid deposition and minimal new bone formation, with most of the defect space occupied by fibrovascular connective tissue. This pattern reflects the delayed and incomplete spontaneous healing typically observed in critical-size bone defects in the absence of osteoinductive stimuli or grafting materials [17,18].

In contrast, defects treated with rhBMP-2 exhibited clear signs of active osteogenesis, including early osteoid deposition, alignment of osteoblast-like cells along the margins of the defect, and progressive new bone formation. With increasing healing time, these regenerative features became more evident, particularly in the β-TCP + 20 µg rhBMP-2 group, where the defect was largely bridged by continuous mineralized new bone containing numerous osteocytes within lacunae by eight weeks. Although the β-TCP + 10 µg and β-TCP + 5 µg rhBMP-2 groups also demonstrated evident bone repair, the degree of bone organization and mineralization was comparatively less pronounced. These observations suggest that rhBMP-2 enhances osteogenic differentiation and matrix deposition, while the β-TCP scaffold provides an osteoconductive framework that supports cell attachment and bone ingrowth. Similar findings have been demonstrated in studies showing that rhBMP-2 delivered through calcium-phosphate biomaterials accelerates bone formation and improves the structural organization of regenerated bone tissue [19,20,21].

Quantitative analysis of new bone formation further supported the histological observations. At the early two-week interval, the control group exhibited minimal new bone formation, indicating limited spontaneous healing of the defect. In contrast, all rhBMP-2–treated groups demonstrated substantially greater bone formation. These findings are consistent with earlier studies demonstrating osteoinductive growth factors can significantly accelerate early bone regeneration when combined with osteoconductive scaffolds [18,22].

As healing progressed to four and eight weeks, new bone formation increased across all groups, reflecting ongoing bone deposition and maturation. Higher rhBMP-2 doses were associated with greater bone formation throughout the healing period. However, the incremental gain between the 10 µg and 20 µg groups at later time points was less pronounced, and no statistically significant difference was observed between these groups (*p* > 0.05), suggesting a tendency toward a saturation or plateau-like response at higher doses. This observation may be partially explained by the highly vascular and trabecular nature of the bone defect environment, which supports robust endogenous healing and may reduce the apparent dose-dependent effect of BMP-2 [23]. These findings indicate that increasing rhBMP-2 beyond a certain threshold may result in diminishing returns in bone regeneration [15]. This observation is consistent with previous reports demonstrating that BMP-induced bone formation typically increases during early healing stages, reaches a peak, and may subsequently plateau or decline depending on factors such as dose, carrier system, and experimental model [24]. Collectively, these findings support the concept that optimal dosing is critical for maximizing the osteogenic potential of BMP-2 [24,25].

The inflammatory response and granulation tissue formation observed in this study demonstrated clear time-related changes following rhBMP-2 application. At the early stage of healing, the control defects exhibited a more pronounced inflammatory reaction, whereas the rhBMP-2–treated groups showed relatively lower inflammatory activity. This finding suggests that rhBMP-2 may contribute to the modulation of the early inflammatory phase of bone repair [26,27]. Granulation tissue was observed in all groups during the early healing phase, with variable presence among the experimental groups, reflecting the initial reparative stage of bone healing [28].

As healing progressed, inflammatory activity gradually declined in all experimental groups, indicating the normal transition from the inflammatory phase to the proliferative stage of tissue repair. Granulation tissue persisted during this stage but showed a gradual decrease over time, consistent with the progression of healing. The semi-quantitative scores demonstrated a temporal reduction in both inflammation and granulation tissue as healing advanced. Previous studies have suggested that BMP-2 may influence the healing process by promoting angiogenesis and regulating cellular responses within the defect site [29,30].

At later stages of healing, inflammatory cells were minimal across groups, and granulation tissue was further reduced, indicating progression toward the remodeling phase of bone repair. The reduction in both inflammation and granulation tissue is consistent with the normal sequence of tissue regeneration. Similar observations have been reported in previous studies, which demonstrated that BMP-2 may facilitate the progression of bone healing from early inflammatory and proliferative stages toward remodeling and maturation [29,31].

The cellular responses observed in this study further highlight the osteogenic activity of rhBMP-2. The rhBMP-2–treated groups demonstrated an increased presence of osteoblasts and osteocytes during the healing period, suggesting enhanced osteogenic activity and progressive maturation of regenerated bone tissue. These findings are consistent with previous studies demonstrating that BMP-2 promotes the recruitment and differentiation of mesenchymal progenitor cells into osteoblasts through activation of Smad signaling pathways and up-regulation of key osteogenic transcription factors such as RUNX2 and Osterix [29,31,32]. Osteoclasts were also observed in the BMP-2 groups, particularly at later healing intervals, indicating active bone remodeling. Osteoclast-mediated resorption plays an essential role in the transition of newly formed woven bone into mature lamellar bone [31]. Recent studies suggest that BMP-2 regulates both osteoblast differentiation and osteoclast activity, thereby coordinating the balance between bone formation and resorption during bone regeneration [33,34,35,36]. This coordinated cellular activity contributes to the development of structurally organized and mechanically functional bone tissue.

Masson’s trichrome staining further demonstrated a progressive increase in collagen deposition and extracellular matrix maturation with increasing dose during the healing process. Collagen fibers gradually increased in density and organization from week two to week eight in all experimental groups; however, the rhBMP-2–treated defects showed more pronounced collagen deposition and improved fiber alignment compared with the control group. Early healing stages in the control defects were characterized by loosely arranged and immature collagen fibers, whereas BMP-2–treated groups already exhibited moderate collagen deposition and partial bundle organization. As healing progressed, collagen bundles became progressively thicker and more organized, particularly in the β-TCP + 10 µg rhBMP-2 and β-TCP + 20 µg rhBMP-2 groups. These findings indicate that BMP-2 enhances extracellular matrix maturation and promotes earlier structural organization of the regenerating bone matrix. This effect may be attributed to the osteoinductive activity of rhBMP-2, which stimulates osteogenic differentiation and type I collagen synthesis, while β-TCP provides an osteoconductive scaffold that facilitates cell attachment and extracellular matrix deposition within the defect site [37,38]. Previous studies have demonstrated that BMP-2 accelerates collagen matrix formation and promotes maturation of regenerated bone tissue in experimental models of bone repair [38,39,40].

Immunohistochemical analysis of CD31 revealed a distinct time- and dose-dependent effect of BMP-2 on vascularization during bone healing. At week 2, all BMP-2–treated groups exhibited significantly higher vascular expression than the control, with a clear dose-dependent increase (20 µg > 10 µg > 5 µg > control), indicating strong stimulation of early angiogenesis. This effect is consistent with the ability of BMP-2 to enhance early neovascularization through osteoblast-mediated release of angiogenic factors such as VEGF [41] and its established role in accelerating early bone healing [42,43].

At weeks 4 and 8, a similar pattern was observed across both intervals, with an overall reduction in CD31 expression in all groups and a reversal of the early dose-dependent trend, where the control group exhibited the highest vascularization followed by 5 µg, 10 µg, and 20 µg groups. This consistent finding suggests that BMP-2 accelerates the angiogenic process, suggesting earlier vascular maturation, thereby diminishing the initial dose-dependent differences over time, while untreated defects maintain a more prolonged physiological angiogenic response. This pattern reflects the normal temporal progression of angiogenesis during bone healing. Such temporal vascular regression is a recognized feature of bone repair, in which early vascular networks undergo stabilization and reduction during tissue maturation [42].

Mechanistically, these findings support the concept that BMP-2–induced angiogenesis is primarily indirect and mediated through osteogenic cell activation and localized paracrine signaling rather than direct endothelial stimulation [44,45]. Furthermore, the tight coupling between angiogenesis and osteogenesis explains the observed time-dependent response [46], where early vascular enhancement is followed by accelerated maturation in BMP-2–treated groups [45].

Several limitations of this study should be acknowledged. First, although the animals were classified as adults based on age and weight, they may not have reached complete skeletal maturity. It is well established that skeletally immature animals exhibit enhanced bone regenerative capacity, which may lead to an overestimation of the osteoinductive effects of rhBMP-2 compared to fully mature or clinical conditions. Second, the use of relatively small calvarial defects (5 mm) may create a more favorable biological environment for bone healing and may not fully replicate large or compromised clinical defects. Therefore, the regenerative outcomes observed in this study may be overestimated relative to clinical conditions, and caution should be exercised when extrapolating these findings to clinical scenarios involving larger defects and fully mature human patients. Third, the absence of a β-TCP-only control group limits the ability to isolate the independent osteoconductive effect of the scaffold from the osteoinductive effect of rhBMP-2. Therefore, the observed regenerative outcomes may reflect the combined effects of the scaffold and growth factor rather than the specific contribution of rhBMP-2 alone, which should be considered when interpreting the results.

Overall, the histological, histomorphometric, and CD31 immunohistochemical findings of the present study demonstrate that rhBMP-2–loaded β-TCP scaffolds enhance bone regeneration in critical-size defects. Regenerative outcomes improved with increasing BMP-2 dosage, with the β-TCP + 20 µg rhBMP-2 group showing the most pronounced regenerative response. These findings support the interaction between osteogenesis and angiogenesis during bone healing and support the role of rhBMP-2 in promoting both processes when delivered through an osteoconductive scaffold system. Future studies should incorporate three-dimensional imaging techniques such as micro-computed tomography (micro-CT) to provide a more comprehensive evaluation of bone volume and architecture. In addition, the use of additional immunohistochemical markers may help to further elucidate the mechanisms of osteogenesis and angiogenesis. Studies with longer healing periods are also recommended to assess the long-term maturation and stability of the regenerated bone.

## 4. Materials and Methods

### 4.1. Animal Model and Experimental Design

Upon approval of the study protocol by the Ethics Committee of Hawler Medical University, an in vivo experimental study was conducted at Hawler Medical University within the Central Animal Care Facility of the College of Pharmacy, Erbil, Kurdistan Region, Iraq. Eighteen healthy adult male New Zealand White rabbits, aged 12–16 weeks and weighing approximately 2.5–3.0 kg, were used in the study. Although the animals were classified as adults based on age and body weight, complete skeletal maturity in rabbits may occur at a later stage. The use of relatively young animals is common in experimental models due to standardization and availability; however, this may influence the rate of bone regeneration. At each time point, six rabbits were sacrificed, and each rabbit contributed one defect to each treatment group (control, β-TCP + 5 µg rhBMP-2, β-TCP + 10 µg rh BMP-2, and β-TCP + 20 µg rh BMP-2). Each rabbit received all treatments; therefore, statistical analysis accounted for within-animal clustering, and the animal was considered the experimental unit.

Prior to surgery, all animals were acclimatized for one week under controlled laboratory conditions to minimize environmental stress and maintain physiological stability. Under general anesthesia, four standardized circular full-thickness defects, each measuring 5 mm in diameter, were created in the calvarial bone of each rabbit, with two defects on each side of the calvaria, resulting in a total of 72 defects. The four defects in each animal were randomly assigned to the experimental groups: control (left unfilled), β-TCP + 5 µg rhBMP-2, β-TCP + 10 µg rhBMP-2, and β-TCP + 20 µg rhBMP-2.

Synthetic β-tricalcium phosphate (adbone^®^ TCP, Medbone^®^ BioMaterials, Sintra, Portugal), composed of 100% pure β-tricalcium phosphate, was used as the osteoconductive scaffold material. The material consisted of granules measuring 1–2 mm in diameter with approximately 80% interconnected porosity and a compressive strength of approximately 3 MPa.

Recombinant human bone morphogenetic protein-2 (rhBMP-2; Elabscience Biotechnology Inc., Wuhan, China) was supplied in lyophilized form (100 µg/vial; purity > 95%) and reconstituted with 0.5 mL sterile water for injection to yield a stock concentration of 200 µg/mL. After complete dissolution, aliquots of the stock solution were applied dropwise onto 0.1 g of β-TCP granules to obtain final doses of 5, 10, and 20 µg per defect, equivalent to 25, 50, and 100 µL, respectively. The graft materials were gently mixed and left at room temperature for approximately 30 min before implantation. All preparation procedures were performed under aseptic conditions. Throughout the study period, the animals were housed in stainless-steel cages under controlled environmental conditions, including a temperature of 19–21 °C, relative humidity of 45 ± 10%, and a 12 h light/dark cycle. All animals were provided with a standard laboratory pellet diet and had free access to drinking water ad libitum [46,47,48].

### 4.2. Surgical Protocol

**Figure 7:**
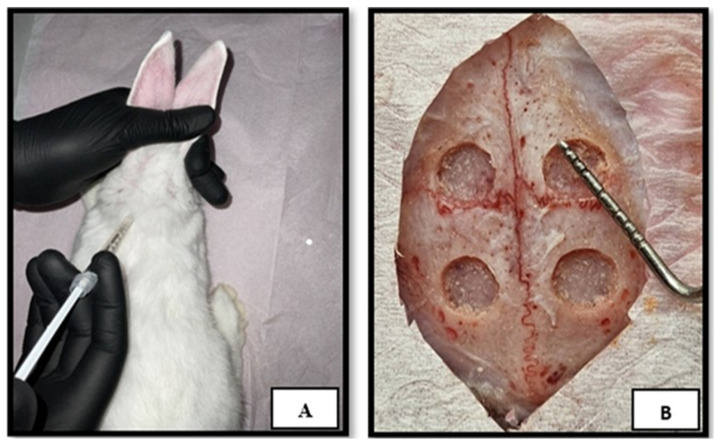
(**A**) Intramuscular anesthesia. (**B**) Creation and measurement of calvarial defects.

All surgical interventions were performed under strict aseptic conditions. Anesthesia was induced by intramuscular injection of ketamine hydrochloride (35 mg/kg) and xylazine (5 mg/kg), as shown in Figure 7A. The calvarial region was then shaved and disinfected with povidone-iodine solution. A midline scalp incision was made, and a full-thickness flap was elevated to expose the calvarial bone. Four standardized full-thickness circular defects, each 5 mm in diameter, were created bilaterally in the parietal bones using a trephine bur under continuous sterile saline irrigation, as shown in Figure 7B. Care was taken to avoid injury to the sagittal suture and underlying dura mater [49].

**Figure 8:**
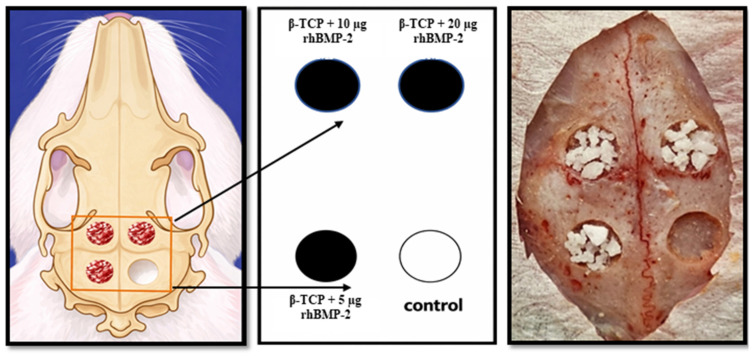
Schematic representation of β-TCP loaded with different doses of BMP-2 applied in 5 mm rabbit calvarial defects.

The defects were treated according to the study design illustrated in Figure 8. The flap was then repositioned and sutured in layers. Postoperative care included intramuscular penicillin (100 mg/kg) once daily for three days and ketorolac tromethamine for analgesia for three days. All animals were monitored daily after surgery.

### 4.3. Histological Evaluation of Rabbit Calvarial Bone Defect

Animals were humanely euthanized at 2, 4, and 8 weeks after surgery, and the calvarial bone specimens were carefully harvested using a dental diamond disc. The samples were fixed in 10% neutral buffered formalin for 48–72 h and subsequently decalcified in 10% formic acid for approximately 2–3 days. Following decalcification, the specimens were dehydrated through graded ethanol solutions, cleared in xylene, and embedded in paraffin wax for histological processing.

Serial sections approximately 5 μm thick were obtained through the center of each defect using a rotary microtome (Leica RM2135, Leica Microsystems, Wetzlar, Germany) [50,51]. The central sections from each block were selected for staining. The sections were stained with hematoxylin and eosin (H&E) for general histological evaluation and Masson’s trichrome for the assessment of collagen deposition and maturation.

Histological evaluation of the stained sections was performed using a light microscope at ×400 magnification. Digital images were captured for qualitative and quantitative analysis of bone formation and tissue organization. The numbers of osteoblasts, osteocytes, and osteoclasts were counted in six randomly selected high-power fields (HPFs). To minimize observer bias, all histopathological assessments were conducted by an experienced histopathologist who was blinded to the experimental groups [52].

### 4.4. Histomorphometric Assessment

Quantitative histomorphometric evaluation of bone regeneration within the defect area was performed using a digital light microscope at ×400 magnification integrated with an image analysis system (Motic, Hong Kong, China; ToupTek, Hangzhou, China; ToupView (×86), version 3.7.4183, 2014). Digital images of microscopic fields covering the entire defect region were captured for analysis.

For quantitative assessment, the region of interest (ROI) was defined as the original defect area corresponding to the 5 mm circular calvarial defect created during surgery. The boundaries of the defect were identified histologically based on the margins of the native cortical bone surrounding the defect.

New bone formation within the ROI was identified according to its histological characteristics and measured using image analysis software. The area of newly formed bone was calculated and expressed as a percentage of the total defect area within the ROI.

Granulation tissue within the defect region was identified based on its histological appearance and evaluated semi-quantitatively using a histological scoring system. The inflammatory response was assessed by counting inflammatory cells, including neutrophils, lymphocytes, and macrophages. Each microscopic field was superimposed with a standardized 16-square grid to facilitate uniform and reproducible cell counting.

### 4.5. Evaluation of Inflammatory Reaction

The severity of the inflammatory response was determined based on the number of inflammatory cells observed at ×400 magnification in all experimental groups [41]. The inflammatory cells were identified according to their morphological characteristics. The inflammatory reaction was graded according to the following criteria:

Score 0: Absence or presence of very few inflammatory cells (0–5), indicating no detectable inflammatory reaction.

Score 1: Between 5 and <25 cells, representing a mild inflammatory reaction.

Score 2: Between 25 and <125 cells, corresponding to a moderate inflammatory reaction.

Score 3: 125 cells or more, indicating a severe inflammatory reaction.

In addition to inflammatory cell counts, the presence of granulation tissue was evaluated semi-quantitatively using a scoring system [53].

Score 0: Weak or minimal expression of the parameter.

Score 1: Moderate expression of the parameter.

Score 2: Strong or intense expression of the parameter.

### 4.6. Special Stains

Masson’s trichrome staining was used to evaluate the distribution and organization of collagen fibers. The extent and pattern of collagen maturation and arrangement were assessed semi-quantitatively according to the following criteria [54]:

Score 0: Predominance of immature, thin collagen fibers arranged in a loose or reticular pattern.

Score 1: Moderate development of collagen fibers forming partially organized bundles.

Score 2: Abundant mature collagen fibers showing compact and parallel organization.

### 4.7. Immunohistochemical Analysis

Formalin-fixed, paraffin-embedded tissue sections (4–5 µm thick) were subjected to immunohistochemical staining for CD31 to evaluate endothelial cell expression and vascularization within the regenerated tissue. The sections were deparaffinized in xylene and rehydrated through graded alcohols, followed by rinsing in distilled water. Heat-induced antigen retrieval was performed using EnVision FLEX Target Retrieval Solution in a PT Link instrument at 97 °C. After washing with EnVision FLEX Wash Buffer, the sections were incubated with a mouse monoclonal anti-CD31 antibody (clone JC70A, Dako/Agilent, Glostrup, Denmark) at room temperature. Subsequently, an HRP-conjugated secondary antibody from the EnVision FLEX detection system was applied according to the manufacturer’s instructions. The antigen–antibody complexes were visualized using 3,3′-diaminobenzidine (DAB) as the chromogen, producing a brown reaction product at sites of CD31 expression. Finally, the sections were counterstained with hematoxylin, dehydrated through graded alcohols, cleared in xylene, and mounted with coverslips for microscopic examination.

The immunostained slides were analyzed using ImageJ software (version 1.47v; National Institutes of Health, Bethesda, MD, USA). A standardized intensity threshold method was applied to identify CD31-positive areas, and the percentage of brown-stained regions was calculated for each specimen. The obtained data were subsequently subjected to statistical analysis to compare vascularization among the experimental groups [55].

Negative controls were included by omitting the primary antibody during the staining procedure. These sections showed no specific staining, confirming the specificity of CD31 immunoreactivity.

### 4.8. Statistical Analysis

Data are presented as mean ± standard deviation (SD). Semi-quantitative ordinal data were analyzed using non-parametric tests and are presented descriptively as mean ± SD. The normality of the data distribution was assessed using the Shapiro–Wilk test. For normally distributed data, differences between treatment groups and healing periods were analyzed using two-way analysis of variance (ANOVA) followed by Tukey’s post hoc test for multiple comparisons. For non-parametric or ordinal data, the Kruskal–Wallis test followed by Dunn’s post hoc test was applied. A *p*-value < 0.05 was considered statistically significant. All statistical analyses were performed using GraphPad Prism software (version 9.0, GraphPad Software, San Diego, CA, USA). Sample sizes were determined by performing a power analysis using G*Power software (version 3.1.9.7, Heinrich Heine University, Düsseldorf, Germany) based on new bone formation measurements obtained from previous studies using rabbit calvarial bone defect models. The statistical parameters were set at a power of 80% (1 − β = 0.80) and a significance level of α = 0.05, with an estimated effect size of 0.40 derived from previously reported experimental data. These power calculations, together with previously reported data from similar calvarial defect experiments, suggested that a sample size of 5–6 animals per time point would be sufficient to detect statistically significant differences between experimental groups.

## 5. Conclusions

Within the limitations of the present study, rhBMP-2–loaded β-tricalcium phosphate scaffolds enhanced bone regeneration in rabbit critical-sized calvarial defects compared with untreated controls. Bone formation, collagen maturation, and vascularization increased with higher rhBMP-2 doses up to a threshold, beyond which no substantial additional benefit was observed. These findings indicate that rhBMP-2 contributes to improved osteogenesis and angiogenesis when combined with an osteoconductive scaffold, although the optimal therapeutic effect appears to occur within a specific dose range rather than increasing indefinitely. However, the clinical relevance of these results is limited by the use of relatively small defect sizes and skeletally immature animals, which may enhance the biological response compared to clinical conditions. In addition, the absence of a β-TCP-only control group restricts the ability to isolate the independent effect of the scaffold. Therefore, further studies using skeletally mature models, larger and clinically relevant defect sizes, and additional control groups are required to validate these findings and optimize the clinical application of rhBMP-2–based regenerative strategies. 

## Figures and Tables

**Table 1:** Quantitative Analysis of new bone formation (%) in Control and β-TCP Loaded with Different Doses of rhBMP-2 in week 2, 4, and 8 (Mean ± SD).

Groups	Week 2 New Bone Formation (%)	Week 4 New Bone Formation (%)	Week 8 New Bone Formation (%)
Control	12.4 ± 3.1	25.7 ± 4.2	38.5 ± 5.0
β-TCP + 5 µg rhBMP-2	18.6 ± 3.8 *	36.2 ± 4.5 *	52.4 ± 6.1 *
β-TCP + 10 µg rhBMP-2	22.9 ± 4.0 **	45.1 ± 5.2 **	65.8 ± 6.7 **
β-TCP + 20 µg rhBMP-2	27.3 ± 4.4 ***	50.6 ± 5.9 ***	72.9 ± 7.2 ***

* *p* < 0.05, ** *p* < 0.01, *** *p* < 0.001.

**Table 2:** Quantitative analysis of CD31-positive vascular area (%) in rabbit calvarial defects treated with β-TCP loaded with different doses of rhBMP-2 at different healing periods.

Groups	Week 2 (%) Mean ± SD	Week 4 (%) Mean ± SD	Week 8 (%) Mean ± SD
Control	18.0 ± 3.0	35.0 ± 4.5	25.0 ± 3.8
β-TCP + 5 µg rhBMP-2	35.0 ± 4.2 *	32.0 ± 4.0 ns	22.0 ± 3.5 ns
β-TCP + 10 µg rhBMP-2	49.0 ± 5.0 *	28.0 ± 3.8 *	18.0 ± 3.0 *
β-TCP + 20 µg rhBMP-2	57.0 ± 5.5 *	25.0 ± 3.5 *	15.0 ± 2.8 *

* *p* < 0.05, ns: not statistically significant (*p* ≥ 0.05).

## Data Availability

The original data presented in this study are included in the article. Further inquiries can be directed to the corresponding author.

## Abbreviations

The following abbreviations are used in this manuscript:

**Abbreviation**	**Definition**
BMP-2	Bone Morphogenetic Protein-2
rhBMP-2	Recombinant Human Bone Morphogenetic Protein-2
β-TCP	Beta-tricalcium phosphate
CSD/CSDs	Critical-size defect/critical-size defects
H&E	Hematoxylin and eosin
HPF/HPFs	High-power field/high-power fields
ROI	Region of interest
SD	Standard deviation
ANOVA	Analysis of variance
HRP	Horseradish peroxidase
DAB	3,3′-Diaminobenzidine
RANKL	Receptor activator of nuclear factor kappa-B ligand
TGF-β	Transforming growth factor beta
VEGF	Vascular endothelial growth factor
RUNX2	Runt-related transcription factor 2
